# Association of Decreased Physical Activity with Rheumatoid Mid-Hindfoot Deformity/Destruction

**DOI:** 10.3390/ijerph181910037

**Published:** 2021-09-24

**Authors:** Takaaki Noguchi, Makoto Hirao, Shigeyoshi Tsuji, Kosuke Ebina, Hideki Tsuboi, Yuki Etani, Shosuke Akita, Jun Hashimoto

**Affiliations:** 1 Department of Orthopaedic Surgery, National Hospital Organization, Osaka Minami Medical Center, 2-1 Kidohigashimachi, Kawachinagano 586-8521, Osaka, Japan; n-takaaki@hotmail.co.jp (T.N.); s.tsuji@ommc-hp.jp (S.T.); akita@ommc-hp.jp (S.A.); junha89@gmail.com (J.H.); 2 Department of Orthopaedic Surgery, Osaka University, Graduate School of Medicine, 2-2 Yamadaoka, Suita 565-0871, Osaka, Japan; y_etani@hotmail.co.jp; 3 Department of Musculoskeletal Regenerative Medicine, Osaka University, Graduate School of Medicine, 2-2 Yamadaoka, Suita City 565-0871, Osaka, Japan; k-ebina@ort.med.osaka-u.ac.jp; 4 Department of Orthopaedic Surgery, Osaka Rosai Hospital, 1179-3 Kita Ward Nagasonecho, Sakai 591-8025, Osaka, Japan; tsubo1155@gmail.com

**Keywords:** physical activity/activity, daily living, midfoot, hindfoot, flatfoot, rheumatoid arthritis

## Abstract

Foot/ankle problems remain important issues in rheumatoid arthritis (RA) patients. Although forefoot deformity generally takes a major place in surgical treatment, concomitant mid-hindfoot deformity is also commonly seen. In this situation, it can be easy to overlook that mid-hindfoot deformity can also induce or exacerbate clinical problems behind the forefoot events. Thus, the relationship between mid-hindfoot deformity/destruction and physical activity/ADL was investigated. Radiographic findings of 101 lower limbs (59 patients) were retrospectively evaluated. Alignment parameters in the lower extremity and joint destruction grade (Larsen grade) were measured. The timed-up-and-go (TUG) test, modified health assessment questionnaire (mHAQ), pain, self-reported scores for the foot and ankle (SAFE-Q), and RA disease activity were investigated to assess clinical status. The relationships among these parameters were evaluated. Subtalar joint destruction was correlated with TUG time (r = 0.329), mHAQ score (r = 0.338), and SAFE-Q: social functioning (r = 0.332). TUG time was correlated with the HKA (r = −0.527), talo-1st metatarsal angle (r = 0.64), calcaneal pitch angle (r = −0.433), M1-M5A (r = −0.345), and M2-M5A (r = −0.475). On multivariable linear regression analysis, TUG time had a relatively strong correlation with the talo-1st metatarsal angle (β = 0.452), and was negatively correlated with calcaneal pitch angle (β = −0.326). Ankle joint destruction was also correlated with TUG time (β = 0.214). Development of structural problems or conditions in mid-hindfoot, especially flatfoot deformity, were related with decreased physical activity in RA patients. Wearing an insole (arch support) as a preventative measure and short foot exercise should be considered from the early phase of deformity/destruction in the mid-hindfoot in the management of RA.

## 1. Introduction

Even with recent improvements in medical treatment for rheumatoid arthritis (RA) providing tight disease control, foot and ankle joint destruction/deformity is often seen. It has been noted that disease activity and remission of RA may result in an underestimation of foot and ankle joint inflammation [1]. Furthermore, it has also been pointed out that there are still many cases of foot and ankle symptoms; thus, self-reported foot and ankle scores should be obtained as additional information to treat RA patients [2]. Indeed, it was reported that self-reported scores revealed that foot problems have a negative impact on foot-related quality of life, affecting general health and physical activity, including for women with foot problems that were not limited to RA [3,4]. Then, conservative foot therapy including footwear and/or exercise should be considered before all else; however, it is reported that there are many RA patients who wear too narrow footwear, as demonstrated in the current situation [5]. Therefore, a surgical approach might often be required for problems induced by progressive foot deformity. In these situations, forefoot deformity generally takes a major place in surgical treatment of the joint with RA, and it occurs within the first three years of RA; approximately 65% of patients have MTP joint involvement [6,7,8,9]. However, at the same time, it has also recently been reported that foot destructive lesions occur in the mid/hindfoot from the early period, within 0–5 years of the RA disease process [10]. Therefore, these factors can mask the fact that mid-hindfoot deformity can also induce or exacerbate clinical problems behind the forefoot events. At the same time, there should be a relationship and interactions between the forefoot and mid/hindfoot deformity during gait and weightbearing in RA patients. In fact, forefoot deformities including hallux valgus are affected by hindfoot/ankle valgus deformity in RA cases [11,12,13,14]. It is also known that hindfoot correction has the potential to reduce ankle joint pain due to realignment of the loading axis [15], suggesting that hindfoot deformity should also have some effects on rheumatoid forefoot and ankle deformities. From these observations, because foot/ankle disorders are known to cause not only pain and gait dysfunction but also falls in RA patients [16,17], it was considered important to evaluate the physical activity and activities of daily living (ADL) of RA patients from the perspective of foot deformity/destruction in the entire (hind/mid/fore) foot with a comprehensive view. In this situation, it is not clear that a mid-hindfoot deformity can cause deterioration of physical activity and ADL. Thus, whether mid-hindfoot deformity/destruction is related to physical activity/ADL was investigated, including parameters of the entire foot. We hypothesized that mid-hindfoot deformity/destruction should have relationships with physical activity/ADL.

## 2. Materials and Methods

### 2.1. Study Design and Patient Population

A retrospective, observational study of 118 lower extremities (59 patients with RA) was performed. As an inclusion criterion, patients needed to have visited a participating hospital (outpatient center) from December 2016 to February 2018 due to knee, foot, or ankle pain/disorders. Of the 118 lower extremities, 17 were excluded because of a past history of some surgery of the lower extremity; this served as an exclusion criterion. The remaining 101 extremities were included in this study, and there was no case with ankylosis of any joints in the lower extremities. Therefore, the calculated sample size was 101. All patients were treated with disease-modifying anti-rheumatic drugs (DMARDs), including methotrexate (MTX) and/or biologics, to control RA disease activity. The patients’ characteristics are shown in Table 1. 

### 2.2. Radiographic Assessment

**Figure 1:**
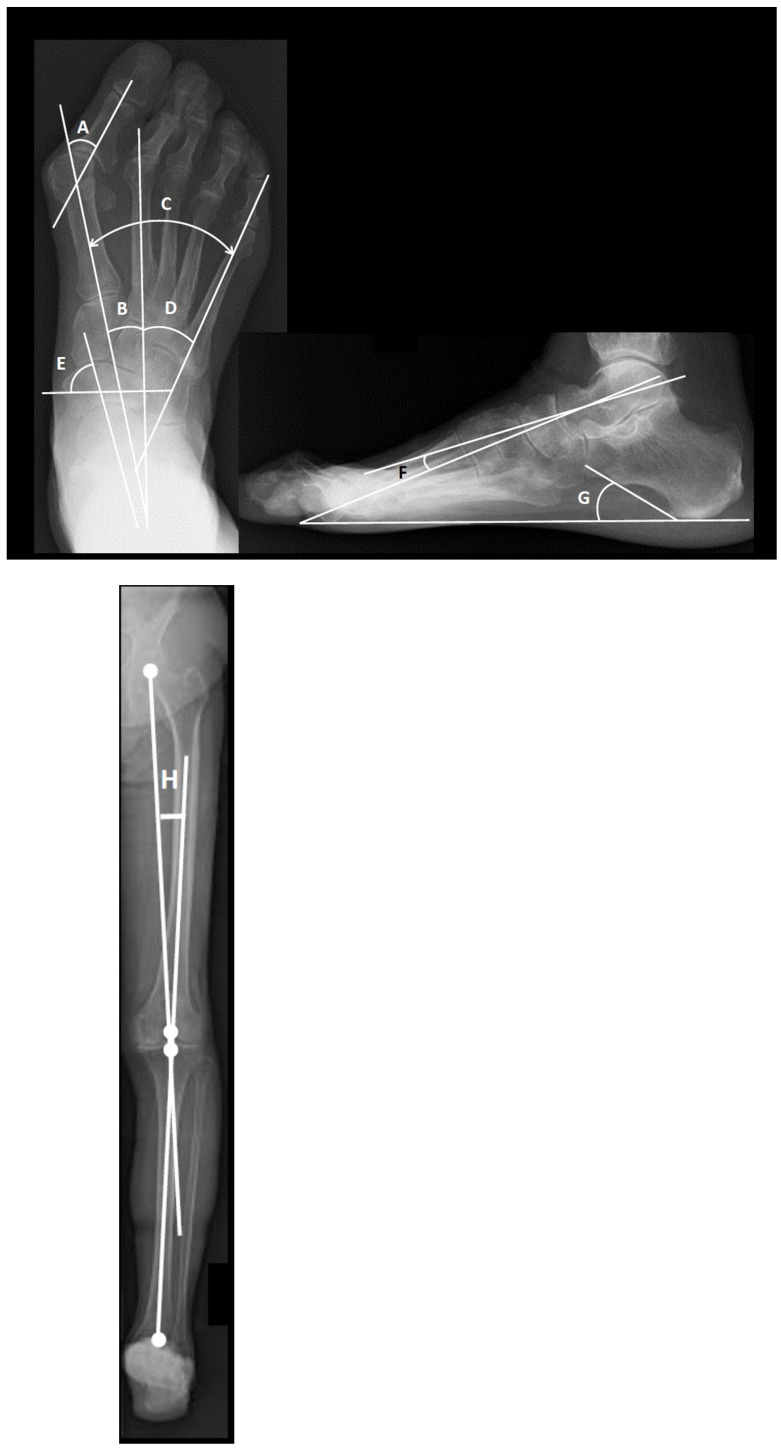
Radiograph to measure parameters of foot deformity. (**A**) Hallux valgus (HV) angle. (**B**) Intermetatarsal angle between the first and second metatarsal bones (M1-M2A). (**C**) Intermetatarsal angle between the first and fifth metatarsal bones (M1-M5A). (**D**) Intermetatarsal angle between the second and fifth metatarsal bones (M2-M5A). (**E**) Pronated foot index (PFI: angle). The PFI is measured as the angle between the short axis of the navicular bone and the long axis of the talus bone (normal ≥65°). (**F**) The talo-1st metatarsal angle (Meary’s angle). (**G**) Calcaneal pitch angle. Radiographs taken in the weight-bearing position. (**H**) Hip–Knee–Ankle angle: Angle between the mechanical axis of the femur and the tibia. The mechanical axis of the femur is the line drawn from the center of the femoral head to the center of the intercondylar notch, whereas the mechanical axis of the tibia is the line connecting the center of the talus to the midpoint of the medial and lateral tibial spine tips.

Dorsoplantar or anteroposterior weightbearing radiographs were taken, and the radiographic assessments were performed as described previously [18]. In brief, as shown in Figure 1, the hallux valgus angle (HVA); the intermetatarsal angles between the first and second metatarsal bones (M1-M2A), between the first and fifth metatarsal bones (M1-M5A), and between the second and fifth metatarsal bones (M2-M5A); Hardy grade [19]; and the pronated foot index (PFI: angle) [18] were measured on dorsoplantar weight-bearing foot radiographs. PFI was measured as the angle between the short axis of the navicular bone and the long axis of the talus bone (normal ≥65°). The talo-1st metatarsal angle (Meary’s angle) [20] and the calcaneal pitch angle were measured on the weight-bearing lateral foot radiographs to evaluate the level of flatfoot deformity. A radiograph of the subtalar joint (modified Cobey method) [21] was used to measure the tibio-calcaneal angle (TCA). A TCA angle ≥2° means a valgus. Loading transmission to the toes is passed through the hip, knee, ankle/hindfoot, midfoot, and forefoot. In this system, knee varus is associated with the development/progression of ankle osteoarthritis [22]. Furthermore, realignment of the knee joint has a potential to change the talar tilt angle, subsequently ameliorate ankle pain, and improve foot/ankle function [23], suggesting that knee alignment affects the loading pattern on the ankle joint. Thus, to check knee alignment, the hip–knee–ankle angle (HKA) [23] was also measured. A positive HKA angle indicates varus alignment of the knee joint. Values of the alignment parameters in this study are shown in Table 2. Evaluations of joint destruction of the hip, knee, ankle, talo-navicular, and subtalar joints were based on Larsen grade classifications [10,24]. As the Larsen grade in the foot was not included in the conventional standard reference, subtalar joint destruction was evaluated using standard references established by Matsumoto et al. [10].

### 2.3. Clinical Assessment

For the clinical assessment, pain was assessed using each patient’s visual analog scale (pVAS) and the doctor’s VAS (dVAS), and RA disease activity was evaluated using the DAS28-CRP score [25]. To evaluate physical activity and static/dynamic balance, the timed-up-and-go (TUG) test was performed [26]. Longer TUG time indicates deterioration of physical activity. To evaluate patient-reported outcomes regarding ADL, the modified Health Assessment Questionnaire (mHAQ) was used [27]. For the clinical assessment, patients also completed a self-administered foot evaluation questionnaire (SAFE-Q) [28].

### 2.4. Statistical Analysis

Pearson’s rank correlation coefficient was used to investigate the correlations of grades of all combinations of radiographic parameters and clinical assessments in this study using single linear regression analysis. Furthermore, to analyze correlation coefficients between TUG time and radiographic/clinical assessment parameters, multivariable linear regression analysis with a forward stepwise procedure was performed. The parameters showing no significant result on single linear regression analysis were excluded. The 95% confidence intervals (CIs) for correlation coefficients were calculated using the Fisher z transformation. Differences with a *P* value of less than 0.05 were considered significant. These data analyses were performed using IBM SPSS Statistics version 22 software (IBM, Armonk, NY, USA).

## 3. Results

### 3.1. Correlations between Knee and Entire Foot Deformities, and Clinical Parameters

In the background of the patient population, as shown in Table 1 and Table 2, correlation analysis was performed. TUG time had correlations with HKA (*r* = −0.527, *p* < 0.001), M1-M5A (*r* = −0.345, *p* < 0.007), M2-M5A (*r* = −0.475, *p* < 0.001), the talo-1st metatarsal angle (*r* = 0.64, *p* < 0.001), and the calcaneal pitch angle (*r* = −0.433, *p* < 0.001). The mHAQ score had correlations with HKA (*r* = −0.256, *p* = 0.013), the talo-1st metatarsal angle (*r* = 0.232, *p* = 0.025), and the calcaneal pitch angle (*r* = −0.233, *p* = 0.024). The SAFE-Q physical functional score had a correlation with HKA (*r* = 0.41, *p* = 0.009). The SAFE-Q social functioning score had a correlation with M1-M5A (*r* = −0.4, *p* = 0.006) (Table 3). 

On multivariable linear regression analysis (Table 4), TUG time had significant correlations with HKA (β = −0.277), the talo-1st metatarsal angle (β = 0.452), the calcaneal pitch angle (β = 0.326), and M2-M5A (β = −0.256) as independent factors. The mHAQ had a correlation with the calcaneal pitch angle (β = −0.29). The social functioning score (SAFE-Q) had a significant correlation with M1-M2A (β = −0.347).

### 3.2. Correlations between Destruction of Each Joint and Clinical Parameters

In the background of the patient population, as shown in Table 1 and Table 2, correlation analysis was performed. TUG time had correlations with the destruction grade of the knee joint (*r* = 0.286, *p* = 0.025), ankle joint (*r* = 0.252, *p* = 0.05), and subtalar joint (*r* = 0.329, *p* = 0.01). The mHAQ score had correlations with the destruction grade of the knee joint (*r* = 0.249, *p* = 0.016), talo-navicular joint (*r* = 0.234, *p* = 0.023), and subtalar joint (*r* = 0.338, *p* = 0.001). The SAFE-Q physical functioning score had a correlation with hip joint destruction (*r* = 0.37 *p* = 0.02), and the SAFE-Q social functional score had a correlation with subtalar joint destruction (*r* = 0.332, *p* = 0.039) (Table 3). On the multivariable linear regression analysis (Table 4), TUG time had a correlation with the grade of ankle joint destruction (β = 0.214). On the other hand, the mHAQ score and SAFE-Q physical/social score had no significant correlations with the destruction of any joint.

### 3.3. Correlations among Clinical Parameters

TUG time had correlations with age (*r* = 309, *p* = 0.015), pVAS (*r* = 0.479, *p* = 0.003), and mHAQ (r = 0.586, *p* < 0.001). The mHAQ score had correlations with pVAS (*r* = 0.447, *p* < 0.001), dVAS (*r* = 0.326, *p* = 0.001), CRP (*r* = 0.366, *p* < 0.001), the DAS28-CRP score (*r* = 0.558, *p* < 0.001), and TUG time (*r* = 0.581, *p* < 0.001). The SAFE-Q physical/social score had no significant correlations with any clinical parameters (Table 3). On multivariable linear regression analysis (Table 4), TUG time had correlations with age (β = 0.29) and mHAQ (β = 0.281). The mHAQ score had correlations with pVAS (β = 0.288) and dVAS (β = 0.328). The SAFE-Q physical functioning score had a correlation with the SAFE-Q social functioning score (β = 0.755). The SAFE-Q social functioning score was also correlated with the SAFE-Q physical functioning score (β = 0.756) (Table 4).

### 3.4. Correlations among Joints with Destruction

As shown in Table 5, the grade of ankle joint destruction was significantly correlated with talo-navicular joint destruction (β = 0.423). Talo-navicular joint destruction was also significantly correlated with ankle joint destruction (β = 0. 611). Subtalar joint destruction grade was correlated with ankle joint destruction (β = 0.437).

## 4. Discussion

In this study, it was found that the pes planovalgus (flat foot) deformity can cause deterioration of physical activity because TUG time had a relatively strong correlation with the talo-1st metatarsal angle (β = 0.452) and a relatively weak correlation with the calcaneal pitch angle (β = −0.326) on multivariable linear regression analysis (Table 4). As a recent report described foot destructive lesions occurring in the mid/hindfoot from the early period of the RA disease process [10], it may be plausible that mid-hindfoot deformities of the pes planovalgus deformity occurred in the early period. Thus, wearing an insole (arch support) as a preventive measure should be considered from the early phase of deformity/destruction in the mid-hindfoot. Short foot exercise for flat foot should also be recommended because of their contribution to improving the dynamic balance ability of the leg and maintaining the medial longitudinal arch [29]. These points should be thoroughly addressed by all rheumatologists and orthopedic surgeons to manage RA patients before physical ability deteriorates in order to extend healthy life expectancy. If such conservative therapy is unable to stop the progression of deformity and deterioration of physical activity, corrective surgery for flatfoot should help improve it in patients with RA. A network system between rheumatologists and foot surgeons is considered very important. As the pattern of loading transmission to the foot would be changed if the knee and/or hindfoot were deformed in the process of RA, knee alignment (HKA) was also included in this investigation. Knee valgus deformity should be kept in mind as being able to cause deterioration of physical activity because TUG time had a relatively weak and negative correlation with HKA (β = −0.277) (Table 4); furthermore, valgus knee deformity is more often seen in RA than in osteoarthritis (OA). It is considered that quadriceps muscle training and a knee support might also be strongly recommended by rheumatologists.

Concerning the association of ADL and physical activity with joint destruction in the lower extremity, although ankle joint destruction was significantly correlated with TUG time on multivariable linear regression analysis, subtalar and talo-navicular joint destruction was also correlated with TUG time on single linear regression analysis. Subtalar joint destruction was also correlated with the SAFE-Q social functioning score. At the same time, the grades of ankle joint destruction including the ankle, subtalar, and talo-navicular joints had significant correlations with each other (Table 5), suggesting that inflammation/synovitis had communicated between neighboring joints, including the ankle, subtalar, and talo-navicular joints. From these observations, it is important to keep in mind that mid-hindfoot joint destruction could cause deterioration of physical activity from the early phase of RA. In this regard, it is also recommended that an arch support be worn. As there were no significant correlations between pVAS/dVAS and deformity/destruction parameters of the foot/ankle in the present study, missing or leaving deformities in the lower body that could impair ADL and physical mobility should be avoided. Adequate timing of surgery for destructive flatfoot deformities might be considered if conservative therapy does not stop the destruction. As age also showed a significant relationship with increased TUG time (Table 4), strengthening medical treatment, wearing arch supports, and surgery for regaining ADL or mobility should be performed before physical activity deteriorates in order to extend healthy life expectancy. Though, on multivariable linear regression analysis, TUG time and mHAQ had no correlation, each parameter was correlated on single linear regression analysis and the mHAQ score had correlations with both pVAS and dVAS. Thus, when the patient complains of pain, a check of physical activity and ADL should always be conducted.

With regard tos the limitations and weaknesses of this study, because this was a cross-sectional study, ADL, physical activity, and each malalignment parameter should be evaluated over time in the future. Furthermore, the flexion angles of the hip and knee joints are also considered important because pelvic tilt has effects on the hip flexor/extensor muscles; subsequently, knee joint status is also changed during weight-bearing. The strength of muscles from the lumbar spine to the foot also needs to be evaluated. Furthermore, a simple index of flatfoot deformity should be established for physicians to manage RA patients in their busy daily medical practice. On the other hand, a strength of this study is that it is the first report to describe the foot problems of RA patients in terms of joint deformity/destruction. Furthermore, in the situation that forefoot deformity is noticeable in RA patients, it was also reported that the mid-hindfoot should be taken into account in the management of RA.

## 5. Conclusions

In conclusion, increased valgus knee deformity and flatfoot deformity were correlated with prolonged TUG time. Flatfoot deformity was related to the deterioration of physical activity. Mid-hindfoot joint destruction was associated with prolonged TUG time. Wearing an insole (arch support) as a preventative measure and short foot exercise might also be considered from the early phase of deformity/destruction in the mid-hindfoot in the management of RA.

## Figures and Tables

**Table 1:** Characteristics of patients with RA and ADL/physical mobility status.

	*N* = 59
Age (y)	67.1 ± 12.0
Male–female ratio (n)	0:59
Disease duration (y)	21.3 ± 13.1
Weight (kg)	49.2 ± 8.5
BMI (kg/m^2^)	21.5 ± 3.44
Steinbrocker stage (I, II, III, IV) (n)	0, 7, 12, 40
Steinbrocker class (I, II, III, IV) (n)	0, 36, 23, 0
DAS28-CRP score	2.77 ± 0.85
Prednisolone usage (%)	50.8
Prednisolone dosage (mg/day)	1.9 ± 2.4 (0–10)
Methotrexate usage (%)	67.8
Biologics usage (%)	23.7
Biologics used (n)	TCZ: 5, IFX: 3, ETN: 3, ABT: 3
SAFE-Q (Physical functioning/Social functioning)	62.55 ± 19.11/63.95 ± 25.40
mHAQ score (points)	0.71 ± 0.7
TUG average time (seconds)	14.9 ± 12.1

Data are presented as means ± SD unless otherwise noted. BMI: body mass index, SAFE-Q: Self-Administered Foot Evaluation Questionnaire, mHAQ: modified Health Assessment Questionnaire, TUG: timed-up-and-go test, TCZ: tocilizumab, IFX: infliximab, ETN: etanercept, ABT: abatacept.

**Table 2:** Values of alignment/destruction parameters.

HKA (hip–knee–ankle) angle (°)	0.4 ± 5.3 (−15–8)
Tibiocalcaneal angle (°)	6.3 ± 5.6 (−10–28)
Pronated foot index (°)	73.4 ± 12.4 (40–101)
Talo-1^st^ metatarsal angle (°)	14.0 ± 11.5 (−17–51)
Calcaneal pitch angle (°)	15.4 ± 6.4 (−2–31)
Intermetatarsal angle between 1st and 2nd metatarsal bones (°)	11.9 ± 5.0 (2–30)
Intermetatarsal angle between 1st and 5th metatarsal bones (°)	32.2 ± 6.8 (13–47)
Intermetatarsal angle between 2nd and 5th metatarsal bones (°)	20.3 ± 5.7 (7–33)
Hallux valgus angle (°)	30.7 ± 18.2 (−4–66)
Hip Larsen (0, I, II, III, IV, V) (n)	75, 15, 8, 1, 2, 0
Knee Larsen (0, I, II, III, IV, V) (n)	73, 11, 3, 5, 6, 3
Ankle Larsen (0, I, II, III, IV, V) (n)	26, 37, 5, 12, 16, 5
Talo-navicular Larsen (0, I, II, III, IV, V) (n)	20, 31, 15, 11, 20, 4
Subtalar Larsen (0, I, II, III, IV, V) (n)	44, 31, 11, 9, 3, 3

Data are presented as means ± SD (range).

**Table 3:** Correlation coefficients between physical activity/ADL and other parameters. (**A**) Correlation coefficients between TUG time and other parameters. (**B**) Correlation coefficients between mHAQ and other parameters. (**C**) Correlation coefficients between SAFE-Q (physical functioning) and other parameters. (**D**) Correlation coefficients between SAFE-Q (social functioning) and other parameters.

(A)							
	HKA Angle	TCA	PFI	Talo-1st Metatarsal Angle	Calcaneal Pitch Angle	HVA	M1-M5A
TUG time	r = −0.527 (*p* < 0.001)	r = −0.082 (*p* = 0.532)	r = −0.024 (*p* = 0.857)	r = 0.64 (*p* < 0.001)	r = −0.433 (*p* < 0.001)	r = −0.054 (*p* = 0.681)	r = −0.345 (*p* = 0.007)
	Knee Larsen	Subtalar Larsen	pVAS	mHAQ	SAFE-Q Physical functioning	SAFE-Q Social functioning	
TUG time	r = 0.286 (*p* = 0.025)	r = 0.329 (*p* = 0.01)	r = 0.479 (*p* = 0.003)	r = 0.586 (*p* < 0.001)	r = 0.061 (*p* = 0.821)	r = 0.194 (*p* = 0.472)	
(**B**)							
	**HKA** **Angle**	**Talo-1st Metatarsal Angle**		**Calcaneal Pitch Angle**	**Knee** **Larsen**	**Talo-Navicular Larsen**	**Subtalar Larsen**
mHAQ	r = −0.256 (*p* = 0.013)	r = 0.232 (*p* = 0.025)		r = −0.233 (*p* = 0.024)	r = 0.249 (*p* = 0.016)	r = 0.234 (*p* = 0.023)	r = 0.338 (*p* = 0.001)
	pVAS	dVAS		CRP	DAS28-CRP	TUG	
mHAQ	r = 0.447 (*p* < 0.001)	r = 0.326 (*p* = 0.001)		r = 0.366 (*p* < 0.001)	r = 0.558 (*p* < 0.001)	r = 0.581 (*p* < 0.001)	
(**C**)							
	**Disease** **Duration**	**HKA** **Angle**		**Talo-1st Metatarsal Angle**		**HVA**	**M1-M5A**
SAFE-Q (Physical functioning)	r = −0.444 (*p* = 0.034)	r = 0.41 (*p* = 0.009)		r = −0.076 (*p* = 0.645)		r = −0.042 (*p* = 0.799)	r = −0.203 (*p* = 0.214)
	Hip Larsen	Subtalar Larsen		mHAQ		TUG	
SAFE-Q (Physical functioning)	r = −0.37 (*p* = 0.02)	r = 0.296 (*p* = 0.068)		r = −0.11 (*p* = 0.636)		r = 0.061 (*p* = 0.821)	
(**D**)							
		**Disease** **Duration**		**HKA** **Angle**		**Talo-1st Metatarsal Angle**	**HVA**
SAFE-Q (Social functioning)		r = −0.317 (*p* = 0.14)		r = 0.266 (*p* = 0.102)		r = −0.101 (*p* = 0.54)	r = −0.239 (*p* = 0.143)
		M1-M5A		Subtalar Larsen		mHAQ	TUG
SAFE-Q (Social functioning)		r = −0.4 (*p* = 0.012)		r = 0.332 (*p* = 0.039)		r = −0.195 (*p* = 0.396)	r = 0.194 (*p* = 0.472)

TUG: timed-up-and-go test, HKA: hip–knee–ankle, TCA: tibiocalcaneal angle, PFI: pronated foot index, HVA: hallux valgus angle, M1-M2A: intermetatarsal angle between the first and second metatarsal bones, M1-M5A: intermetatarsal angle between the first and fifth metatarsal bones, pVAS: patient’s visual analog scales, mHAQ: modified Health Assessment Questionnaire, SAFE-Q: self-administered foot evaluation questionnaire.

**Table 4:** Correlation coefficients between physical activity/ADL and other parameters on multivariable linear regression analysis. (**A**) Correlation coefficients between TUG time and other parameters. (**B**) Correlation coefficients between mHAQ and other parameters. (**C**) Correlation coefficients between SAFE-Q (Physical functioning) and other parameters. (**D**) Correlation coefficients between SAFE-Q (Social functioning) and other parameters.

(A)			
	β	95% CI	*p* value
Age	0.29	0.087–0.326	0.001
HKA angle	−0.277	−0.77–−0.194	0.002
M2-M5A	−0.256	−0.806–0.108	0.011
Ankle Larsen	0.214	0.535–3.286	0.007
Talo-1st metatarsal angle	0.452	0.183–0.604	<0.001
Calcaneal pitch angle	−0.326	0.187–0.92	0.004
mHAQ	0.281	1.88–7.401	0.001
(**B**)			
	β	95% CI	*p* value
Calcaneal pitch angle	−0.29	−0.051–−0.009	0.006
pVAS	0.288	0.019–0.125	0.009
dVAS	0.328	0.053–0.226	0.002
TUG	0.297	0.005–0.031	0.007
(**C**)			
	β	95% CI	*p* value
HKA angle	0.185	0.095–1.799	0.03
Hip Larsen	0.189	0.657–9.521	0.026
SAFE-Q (Social functioning)	0.755	0.454–0.715	<0.001
(**D**)			
	β	95% CI	*p* value
M1-M2A	−0.347	−2.665–0.988	<0.001
Subtalar Larsen	0.17	0.18–8.938	0.042
SAFE-Q (Physical functioning)	0.756	0.767–1.178	<0.001

Multivariable linear regression analysis with a forward stepwise procedure was performed to analyze correlation coefficients. TUG: timed-up-and-go test, ADL: activities of daily living, HKA: hip–knee–ankle, M2-M5A: intermetatarsal angle between the second and fifth metatarsal bones, mHAQ: modified Health Assessment Questionnaire, pVAS: patient’s visual analog scales, dVAS: doctor’s visual analog scales, SAFE-Q: self-administered foot evaluation questionnaire, M1-M2A: intermetatarsal angle between the first and second metatarsal bones.

**Table 5:** Correlation coefficients between mid-hindfoot joint destruction and other parameters on multivariable linear regression analysis. (**A**) Correlation coefficients between Ankle Larsen and other parameters. (**B**) Correlation coefficients between Talo-navicular Larsen and other parameters. (**C**) Correlation coefficients between Subtalar Larsen and other parameters.

(A)			
	β	95% CI	*p* value
Talo-navicular Larsen	0.423	0.114–0.684	0.007
(**B**)			
	β	95% CI	*p* value
Ankle Larsen	0.611	0.352–1.297	0.002
(**C**)			
	β	95% CI	*p* value
Ankle Larsen	0.437	0.033–0.711	0.033

Multivariable linear regression analysis with a forward stepwise procedure was performed to analyze correlation coefficients.

## Data Availability

Not applicable.
